# Navigating biosafety regulatory frameworks for genetic engineering in Africa: a focus on genome editing and gene drive technologies

**DOI:** 10.3389/fbioe.2024.1483279

**Published:** 2024-10-24

**Authors:** Tilahun Rabuma, Felix Moronta-Barrios, Wendy Craig

**Affiliations:** ^1^ Department of Biotechnology, College of Natural and Computational Science, Wolkite University, Wolkite, Ethiopia; ^2^ Regulatory Science Group, International Centre for Genetic Engineering and Biotechnology, Trieste, Italy

**Keywords:** biosafety regulations, emerging biotechnologies, regulatory frameworks, Africa, genome editing, gene drive

## Abstract

Genome editing and gene drive technologies are increasingly gaining attraction in Africa, with researchers exploring their potential applications in agriculture, health and the environment. Acknowledging that robust regulatory frameworks are crucial in facilitating the development and utilization of these technologies, informed decision-making is, however, being impeded by the fragmented information availability and readiness of regulatory authorities on the continent.

**Objectives:**

This study investigates the regulatory frameworks governing genome editing and gene drive technologies in African countries, identifies common regulatory challenges and proposes actionable solutions.

**Methods:**

Primary data were collected through questionnaires and complemented by analysing existing biosafety regulations from online databases and scientific literature.

**Results:**

Our findings suggest that while a few African countries have recently updated their regulatory frameworks, many are still under discussion. Challenges to development and implementation include limited resources, expertise, awareness, and public resistance.

**Conclusion:**

The findings underscore the urgent need for further development in regulatory capacities. By shedding light on these challenges, our study could provide African regulators with valuable insights to guide the formulation of effective regulatory frameworks. Such frameworks are essential for harnessing the potential of genome editing and gene drive technologies while safeguarding human health and the environment in Africa.

## 1 Introduction

Genome editing is the ability to make precise changes to DNA sequences within organisms using engineered nuclease enzymes to cut and replace existing DNA segments (Fridovich-Keil, 2024). Among the numerous genome-editing methods now available, CRISPR/Cas-based genome editing stands out as the most convenient, efficient, precise and widely used genome editing tool (Knott and Doudna, 2018; Li et al., 2022; Matsumoto and Nomura, 2023). Furthermore, CRISPR/Cas-based gene drive systems enable the manipulation of self-propagating genetic elements, which are passed on to offspring at frequencies surpassing Mendelian inheritance (Bier, 2022). Thus, gene drives bias inheritance patterns by increasing their prevalence in successive generations (Alphey et al., 2020).

In Africa, genome editing and gene drive technologies hold promise for addressing pressing challenges in agriculture, health and the environment. For instance, the increasing food demand, exacerbated by factors such as climate change, diseases, and limited access to fertilizers and agrochemicals, necessitates innovative solutions. Genome editing technology is another approach that offers avenues to enhance agricultural productivity by, for instance, developing drought tolerance (Osakabe et al., 2016; Sami et al., 2021; Shelake et al., 2022), disease resistance (Karmakar et al., 2022), salt tolerance (Saradadevi et al., 2021; Shelake et al., 2022), and nutritional improvement (Ku and Ha, 2020; Nagamine and Ezura, 2022).

Likewise, the escalating prevalence of insect-borne diseases like malaria in tropical and subtropical regions of Africa underscores the urgency of novel public health interventions. Despite various control strategies, African countries continue to grapple with the highest malaria burden globally (Ombogo, 2023). In pursuit of the ambitious goal of malaria elimination by 2030, the World Health Organization (WHO) has prioritized gene drive mosquitoes as a transformative technology (Wamba, 2023), which represents a promising new tool for the elimination of malaria and other mosquito-borne diseases (North et al., 2020; Metchanun et al., 2022).

Researchers are increasingly drawn to gene drive technologies due to their potential as highly effective, cost-efficient, and enduring solutions (Bier, 2022; James et al., 2018; James et al., 2023). However, the adoption of genome editing and gene drive technologies highly depends on government regulation in each country (Jenkins et al., 2021). Such regulation plays a crucial role in determining whether approval is necessary and thus given for the development and commercialization of these products (Entine et al., 2021). Moreover, regulatory frameworks serve to safeguard human health and the environment while fostering public trust and legal certainty for research institutions and industries (Zawedde et al., 2018; DiversityS. O. T. C. O. B., 2000).

The global regulatory landscape for genome-edited products is evolving rapidly (Tripathi et al., 2022). While some countries have swiftly adapted legislation or regulatory frameworks to support genome editing, others remain in the policy formulation stages (Jenkins et al., 2021), and some still classify these products as Genetically Modified Organisms (GMOs) (Hundleby and Harwood, 2022). Whether genome-edited products are exempt from GMO regulations often depends on the specific genome-editing techniques used. For instance, certain gene editing methods, such as site-directed nucleases (SDNs), including SDN-1, which induces gene disruptions through insertions or deletions, and SDN-2, which uses homologous templates for gene correction or modification, are fully exempted in some countries (Wolt et al., 2015). In contrast, SDN-3, which involves inserting larger DNA elements or foreign genes, is typically treated as a GMO (Vora et al., 2023). Countries like Argentina, Australia, Brazil, Chile, India, Kenya, Nigeria, Paraguay, Russia, and the USA have exempted genome-edited plants from GMO regulations, while China and the UK follow simplified GMO regulations. The EU, New Zealand and South Africa, however, regulate genome-edited products as GMOs, and in many countries, proper regulations or discussions are still lacking (Friedrichs et al., 2019; Schmidt et al., 2020; Vora et al., 2023).

Although regulatory frameworks for GMOs have been established in many African countries following their adoption of the Cartagena Protocol on Biosafety (Akinbo et al., 2021; Quemada, 2022), several countries are still working to effectively manage modern biotechnology and implement national biosafety frameworks for GMOs (Komen et al., 2020). Furthermore, most African countries do not have adequate regulatory frameworks specifically tailored to regulate genome editing and gene drive technologies regulatory oversight (Masehela and Barros, 2023). A well-established GMO regulatory framework can be a logical departure point when contemplating genome-editing governance (Abkallo et al., 2024). In this regard, efforts have been made to incorporate genome editing products into the existing GMO biosafety regulatory frameworks on a case-by-case basis; however, concerns remain about the overly restrictive nature of regulations concerning the introduction and development of genome editing and gene drive products in Africa (Ongu et al., 2023). Additionally, existing laboratory biosafety and biosecurity review processes may not effectively address the unique challenges posed by gene drive research and its components (Millett et al., 2022). Uncertainties such as limited access to laboratories, equipment and reagents, a shortage of trained professionals for molecular biology work, and a low rate of returnees among the trained professionals working internationally have significantly negatively impacted genome editing research and development in Africa (Abkallo et al., 2024), which, in turn, has also affected the development of regulatory frameworks for genome editing and gene drive technologies.

Concerns also arise regarding the potential dispersion and persistence of gene drive transgenes beyond the release area, posing challenges to their regulation under existing GMO regulatory frameworks (Gene Drives on the Horizon, 2016; Programme, 2021). Furthermore, the lack of mitigation and traceability strategies (Noble et al., 2018), together with limited experience in risk assessment, exacerbates regulatory uncertainties (AGBC, 2022). Responsible field-based gene drive research also raises significant concerns (Thizy et al., 2020), further complicating regulatory efforts. As a result, the regulatory status of genome editing and gene drive technologies in Africa remains uncertain, raising questions about the adequacy of existing frameworks and the need for new or updated regulatory frameworks (Asquer and Morrison, 2022). However, a few African countries have initiated efforts to incorporate genome editing products into their biosafety regulatory frameworks, such as Nigeria and Kenya (Boluwade and Smith, 2021; Tripathi et al., 2022), while other African countries like Burkina Faso, Mali and Uganda plan to start field trials of gene drive mosquitoes within the next 5–10 years (Hartley et al., 2021).

Given these challenges, an African Union policy consultation advocates for a more enabling and science-based regulatory approach in order to leverage genome editing and gene drive technologies (Komen et al., 2020). Dolezel et al. (2020) suggested a need for a comprehensive examination of current GMO regulatory frameworks to determine their suitability for addressing the potential risks and challenges posed by gene drive applications. A comprehensive analysis is required to shed light on the existing regulatory frameworks that govern these technologies in African countries. The aim of our study was, therefore, to analyze the status of regulatory frameworks for genome editing and gene drive technologies and identify gaps in their development and implementation in African nations. To this end, the regulatory approaches of various African countries were compared and contrasted, common trends and differences were identified, the adaptability of the current regulatory frameworks to emerging technologies was assessed and recommendations for improving biosafety regulations in African countries were formulated.

This serves as a comprehensive update on the current status and challenges facing biosafety regulatory frameworks in African countries concerning genome-edited and gene drive technologies, contributing to the advancement of these technologies on the continent. This information can equip African regulators, policymakers and researchers with valuable insights into the establishment of robust regulatory frameworks for genome editing and gene drive products.

## 2 Methodology

This exploratory study adopted a qualitative approach to investigate the regulatory environment for genome editing and gene drive technologies in Africa. A survey was designed with five distinct sections, combining both closed-ended and open-ended questions, thereby allowing for in-depth insights into the aspects of biosafety regulations. The survey was deployed between 22 July and 07 August 2023. The initial section gathered demographic information and general insights. Subsequently, the survey enquired into core aspects, including the status of biosafety regulatory frameworks for genome editing and gene drive technologies, international collaborations and harmonization efforts, identified gaps and challenges in existing regulations and explored public perceptions along with ethical considerations. The survey targeted Cartagena Protocol national focal points and national competent authorities across 54 African countries, aiming to discern the presence and effectiveness of regulatory frameworks. Additionally, data on national biosafety regulatory status was cross-verified through the database of Biosafety Clearing-House (BCH) and the one curated by the African Biosafety Network of Expertise (ABNE). To enhance the comprehensiveness of our findings, a literature search was conducted to supplement and triangulate the information gathered from the questionnaire and databases, ensuring a robust and multifaceted analysis of the regulatory landscape in Africa.

## 3 Results

### 3.1 The authorization for genetic engineering applications in agriculture

The survey findings reveal varying degrees of authorization for genetic engineering applications across African countries at different developmental stages. For example, some African countries may have authorized only research and development (R&D) and confined field trials (CFTS) activities, as exhibited in DRC, Tunisia and Uganda, while others may have authorized GM crops, including the importation and cultivation for feed and food applications (Table 1). Notably, Eswatini, Kenya and Nigeria have authorized genetic engineering applications at nearly all developmental stages. In contrast, countries such as Ethiopia, Ghana, Mozambique, Uganda and Zambia have limited their authorization to laboratory research and confined field trials. While in countries such as Benin, Côte d'Ivoire, DRC, Gambia, Mali, Nambia, Senegal, Togo, Tunisia and Zimbabwe, authorizations for genetic engineering applications at various stages of development are still lagging compared to other African countries.

**TABLE 1:** The status of authorization for biotechnology applications in African countries according to the survey respondents. Data was collected between 22 July and 07 August 2023.

Countries	R&D^a^	CFT^b^	Open-field trial^c^	Pre-market authorization^d^	Importation^e^		Cultivation^f^	
					Feed	Food	Feed	Food
Benin	−	−	−	−	−	−	−	−
Cote d’Ivoire	−	−	−	−	−	−	−	−
DRC	+	−	−	−	−	−	−	−
Eswatini	+	+	−	+	+	+	+	+
Ethiopia	+	+	+	−	−	−	−	−
Gambia	−	−	−	−	−	−	−	−
Ghana	+	+	+	+	+	+	0	0
Kenya	+	+	−	+	+	+	+	+
Mali	−	−	−	−	−	−	−	−
Mozambique	+	+	−	−	−	−	−	−
Namibia	−	-	−	−	−	−	−	−
Niger	−	-	−	−	−	−	−	−
Nigeria	+	+	−	+	+	+	+	+
Senegal	−	−	−	−	−	−	−	−
Togo	−	−	−	−	−	−	−	−
Tunisia	+	−	−	−	−	−	−	−
Uganda	+	+	−	−	−	−	−	−
Zambia	+	+	−	−	+	+	−	−
Zimbabwe	−	−	−	−	−	−	−	−

(Mark +: denotes yes; -: denotes “No”, 0: denotes no response given).

^a^ Research and Development (R&D): The early-stage activities involving laboratory and greenhouse experiments aimed at investigating the potential and safety of genetic engineering technologies.

^b^ Confined Field Trials (CFTs): Small-scale, controlled experiments in open environments to assess genetically engineered crops while preventing their establishment and spread.

^c^ Open Field Trials: Larger-scale controlled releases of genetically engineered crops into the environment to evaluate their performance under real-world conditions, collect data, and mitigate adverse effects.

^d^ Pre-Market Authorization: The approval process required before genetically engineered crops can be sold or distributed commercially, ensuring they meet safety and regulatory standards.

^e^ Importation: The authorization to bring genetically engineered crops or products containing genetically engineered materials into a country for research, trials, or commercial purposes.

^f^ Cultivation: The stage where genetically engineered crops are grown on a commercial scale following successful authorization for general agricultural use.

### 3.2 Status of regulatory framework for genome editing and gene drive technologies in Africa

According to survey respondents, Benin, Eswatini, Kenya, Nigeria and Uganda have established suitable regulatory frameworks to regulate genome editing and gene drive technologies and their products. In Benin, Eswatini and Uganda, existing biosafety regulations can be applied to regulate genome-edited and gene drive products. Kenya and Nigeria have implemented specific regulations tailored to oversee these technologies. In this sense, Nigeria has amended existing biosafety regulations to encompass genome editing products within governmental safety review and approval procedures. Kenya has also developed a new genome editing-specific regulatory framework that oversees technology use within the safety review and requisite governmental approval. For gene drive technology, the Kenyan respondents replied that existing biosafety regulations would be applied. Survey results further highlight that Eswatini, Nigeria and Uganda adopt a process-based approach to regulating these technologies, while Benin and Kenya employ a product-based approach to regulating genome editing technology (Table 2). In Zambia, on the other hand, the approach to regulation remains under development.

**TABLE 2:** Details status of the regulatory framework for genome editing and gene drive technologies in six African countries.

African countries	Does the country have an approved and published regulatory framework?	Types of genome editing technique				Regulatory framework developed and applied	Regulatory approach triggering regulations	Year of approval and publication of biosafety regulatory framework
		SDN-1	SDN-2	SDN-3	ODM			
Benin	−	Deregulated on a case-by-case basis	Deregulated on a case-by-case basis	Regulated	Deregulated on a case-by-case basis	Existing regulation	Product-based	• Not published • Under discussion
Eswatini	−	Deregulated on a case-by-case basis	Deregulated	Regulated	Deregulated	Existing regulation	Process-based	• Not yet published • Under discussion
Kenya	+	Deregulated on a case-by-case basis	Deregulated on a case-by-case basis	Regulated	Deregulated on a case-by-case basis	New guidelines developed	Product-based	Published in 2022
Nigeria	+	Deregulated on a case-by-case basis	Deregulated on a case-by-case basis	Regulated	Reregulated on a case-by-case basis	Modified pre-existing regulations	Process-based	Published in 2019
Uganda	−	Deregulated on a case-by-case basis	Deregulated on a case-by-case basis	Regulated	Deregulated on a case-by-case basis	Existing regulation	Process-based	Under discussion
Zambia	−	Deregulated on a case-by-case basis (under development)	Regulated on a case-by-case basis	Regulated on a case-by-case basis	Regulated on a case-by-case basis	Under discussion	Under discussion	Under discussion

(Mark +: denotes yes; -: denotes “No”).

**FIGURE 1:**
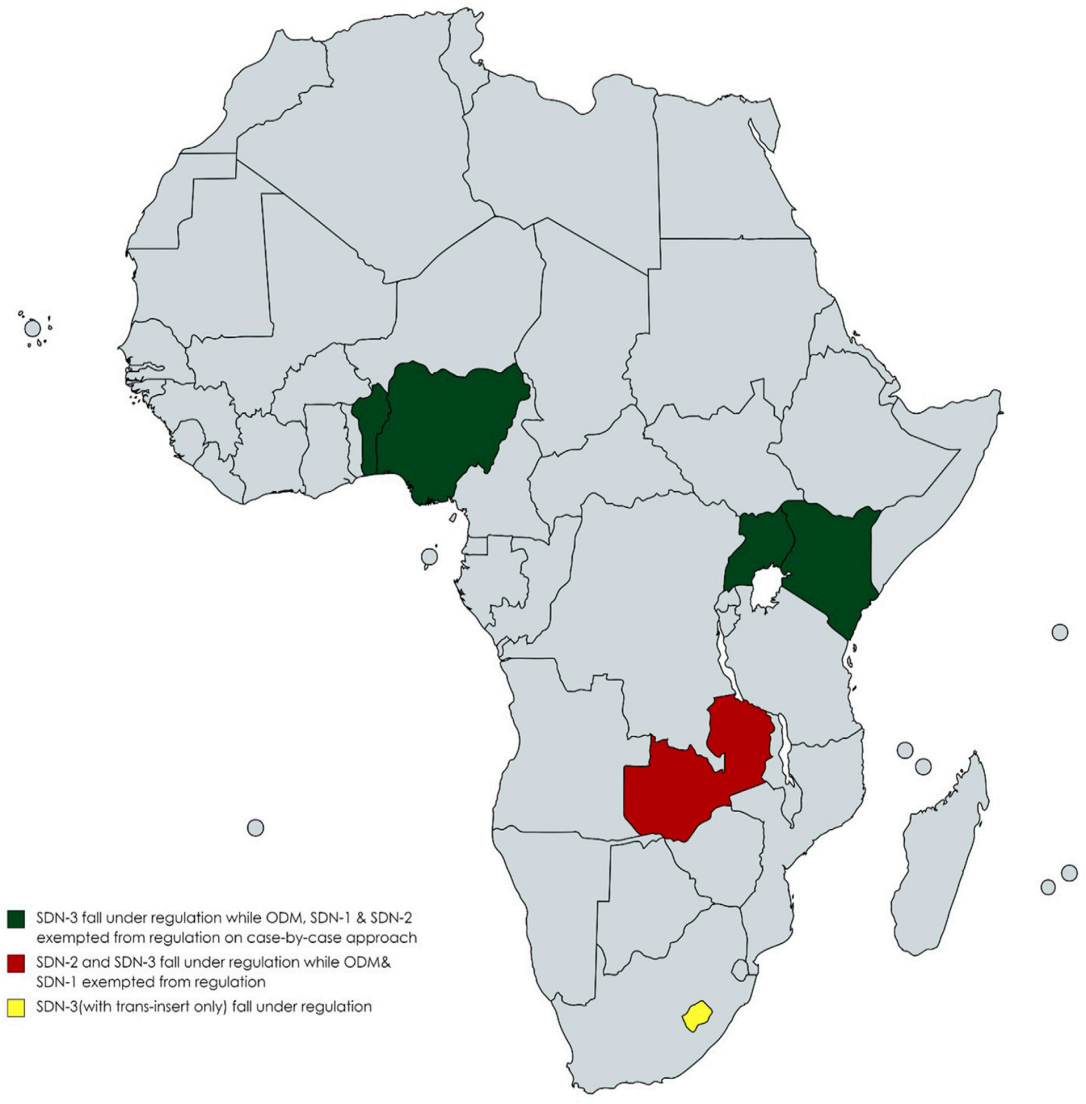
Regulation of genome edited products according to the genome editing technique used by country.

Regarding genome editing techniques, responses from Benin, Kenya, Nigeria and Uganda indicate that those techniques incorporating site-directed nucleases-3 (SDN-3) fall under regulatory oversight, while those utilising site-directed nucleases-1 (SDN-1), site-directed nucleases 2 (SDN-2) and Oligonucleotide-directed mutagenesis (ODM) are exempted from existing regulation oversight on a case-by-case basis (Table 2; Figure 1). Additionally, respondents from Eswatini indicated that SDN-1, SDN-2 and SDN-3(with *cis-insert*) are exempted from GMO regulations on a case-by-case approach, while SDN-3 (with *trans-insert*) are regulated under existing GMO regulations. Similarly, in Zambia, in which the regulatory framework is under development, SDN-2 and SDN-3 categories are expected to fall under existing regulations, while SDN-1 and ODM will be deregulated from GMO regulation on a case-by-case basis.

### 3.3 Challenges

#### 3.3.1 Identified gaps in the implementation of regulatory frameworks

**FIGURE 2:**
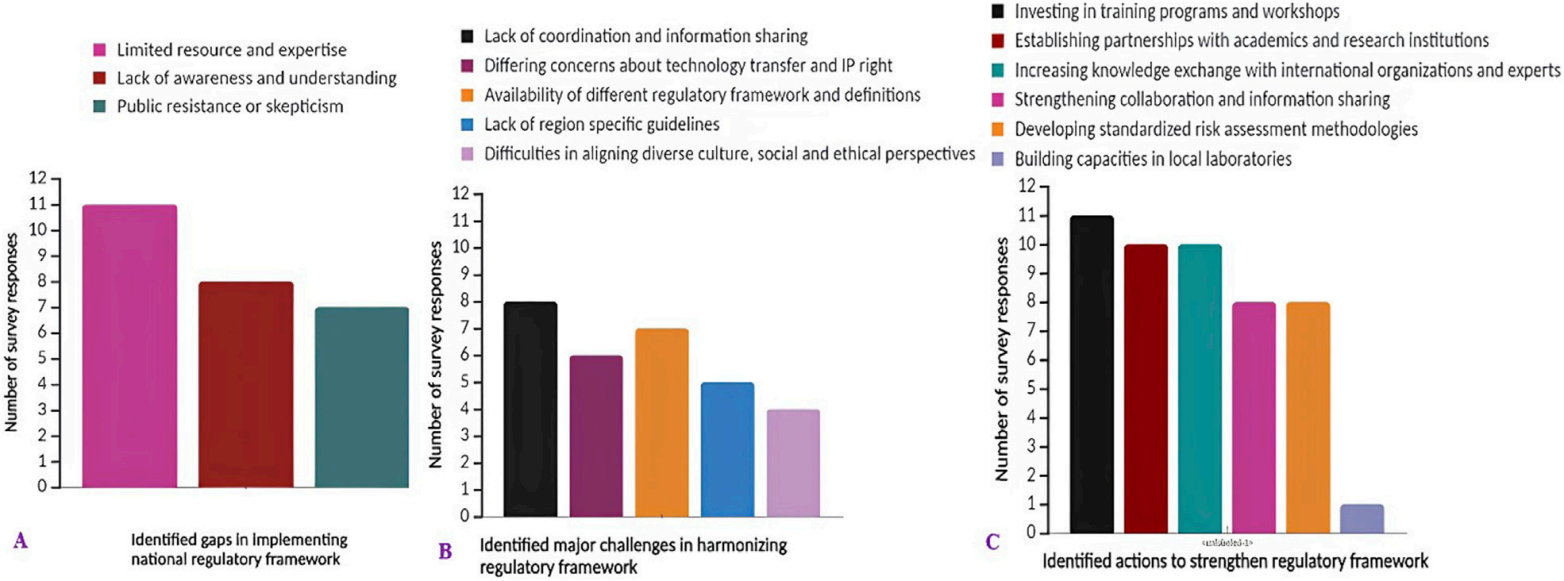
The survey response analysis on: **(A)** identified gaps in the implementation of the national biosafety regulatory framework; **(B)** major challenges or barriers in harmonizing biosafety regulations for genome editing at a regional or international level; **(C)** action needs to strengthen the biosafety regulatory framework for genome editing and gene drive technologies.

Respondents highlighted significant challenges in the implementation of national biosafety regulations for genome editing and gene drives across most African countries. Top among these challenges are limited resources and expertise, coupled with a lack of awareness or understanding of the technologies (Figure 2A). Additionally, the implementation of these regulations faces hurdles in certain countries due to public resistance or scepticism. Notably, respondents from The Gambia and the Democratic Republic of Congo (DRC) indicated gaps in their regulatory framework, with the DRC notably lacking a biosafety law for these technologies. The response from Benin underscores that although lack of awareness or understanding, limited resources and expertise, and public resistance or scepticism exist as gaps in the implementation of the national legal framework for genome editing and gene drive technologies, the issues are so dynamic and difficult to address, suggesting the need for a holistic, comprehensive approach to implementing the framework.

#### 3.3.2 The major challenges in harmonizing biosafety regulatory framework in African countries

The survey findings indicate significant obstacles to aligning the biosafety regulatory framework for genome editing and gene drive technology at a regional or international level in African countries. Foremost among these harmonization challenges is the lack of coordination and information sharing between regulatory authorities. Additionally, differing concerns about technology transfer and intellectual property rights, as well as variations in regulatory frameworks and definitions among African countries, pose considerable hurdles. Aligning diverse cultural, social and ethical perspectives also presents challenges. However, respondents identified this as the least significant barrier to harmonizing the biosafety regulatory frameworks (Figure 2B). Thus, unharmonized regulatory frameworks could potentially hinder the application of genome editing and gene derive technologies and future international trade.

#### 3.3.3 Strengthening regulatory frameworks for genome editing and gene drive technologies

This investigation, drawing input from African biosafety authorities, experts, scientists and civil society, underscores several crucial actions necessary for the development and enhancement of biosafety regulatory frameworks for genome editing and gene drive technologies and products (Figure 2C). Foremost among these actions is the investment in training programmes and workshops aimed at regulators and stakeholders. Furthermore, the analysis identified pivotal actions such as the establishment of partnerships with academic institutions for research and capacity building, along with increasing knowledge-sharing with international organizations and experts. Additionally, strengthening collaboration among African countries and the development of specific, standardized risk assessment methodologies for genome editing and gene drive was indicated as an additional crucial action.

#### 3.3.4 Public perception, resistance and ethical issues

Responses from nine countries, including Benin, the DRC, Ethiopia, Kenya, Mozambique, Nigeria, Tunisia, Uganda, and Zambia, underline significant public resistance and controversies surrounding genome editing and gene drive technologies and products. The reasons cited for this opposition relate to the early stage of development of these technologies and public concerns regarding potential harm to human health and the environment (Table 3). Moreover, respondents from five countries indicated concerns about the potential for unintended consequences or misuse of these technologies. Social media activism emerged as a key driver of resistance and controversies against these technologies. Notably, respondents from Eswatini reported no controversy or public opposition to genome editing or gene drive, while in Nigeria, although controversies exist, there is no apparent public resistance raised through social media activism to prevent activities with the technologies.

**TABLE 3:** Survey analysis on the types of public resistance against genome editing and gene drive technologies.

Countries	Justification for controversies			No controversies about technologies	Is public resistance raised against Genome editing and Gene drive?	Ways of Triggering Public Resistance
	Technologies are early in their development, and thus not enough is known about them	Public believes that products are bad for health	Technologies have the potential to cause unwanted consequences and/or abuse			
Benin	−	−	−	−	+	Social media activism seeks to prevent activities with the technologies
DRC	+	−	+	−	−	−
Eswatini	−	−	−	+	−	−
Ethiopia	+	+	+	−	+	Social media activism seeks to prevent activities with the technologies
Gambia	0	0	0	0	0	0
Kenya	+	−	−	−	+	Social media activism seeks to prevent activities with the technologies
Mozambique	+	+	−	−	0	0
Nigeria	+	+		−	−	0
Tunisia	−	−	+	−	0	0
Uganda	+	−	+	−	+	Social media activism seeks to prevent activities with the technologies
Zambia	−	+	−	−	+	Social media activism seeks to prevent activities with the technologies

(Mark +: denotes yes; -: denotes “No”, 0: denotes no response given).

#### 3.3.5 Improving public perception and attitude

**FIGURE 3:**
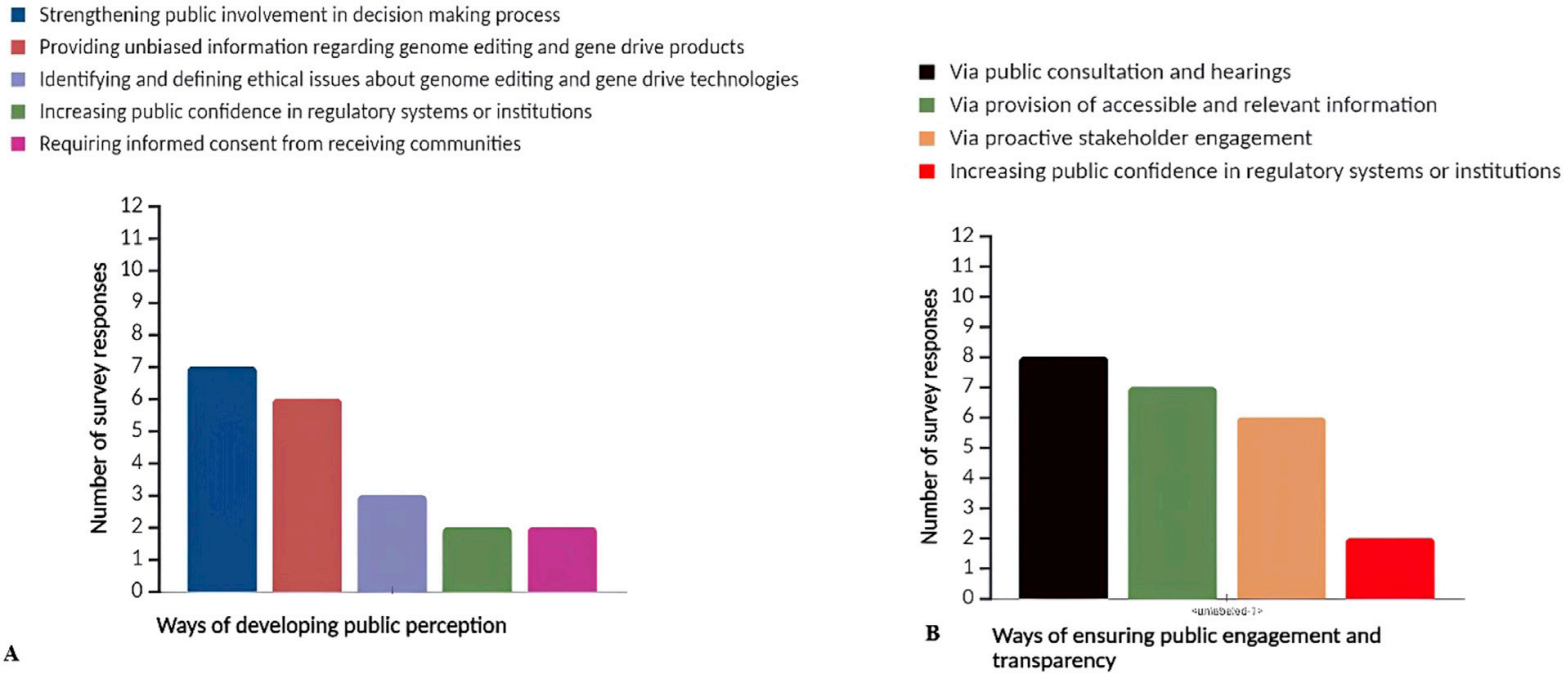
The survey response analysis on: **(A)** ways of public perception and attitude development towards genome editing and gene drive technologies/products; **(B)** ways to ensure public engagement and transparency in the decision-making process towards genome editing and gene drive technologies and products.

Survey responses indicate that public perception can be improved by involving the public more in the decision-making process regarding the release of genome-edited and gene drive products (Figure 3A). Furthermore, providing unbiased information and easily understandable information about these technologies to non-specialists is seen as crucial for improving public perception. Only a few countries mentioned the importance of identifying and defining ethical issues, increasing public confidence in the regulatory systems, and requiring informed consent from recipient communities to support the improvement of public perceptions and attitudes towards genome editing and gene drive technologies.

#### 3.3.6 Ensuring public engagement and transparency

This investigation highlights the importance of public consultation and hearings in the decision-making process regarding genome editing and gene drive technologies (Figure 3B). Additionally, ensuring accessible information about these technologies and proactive stakeholder engagement and transparency in the decision-making process are seen as essential for improving public perception.

### 3.4 Prospective toward developing/drafting the biosafety regulatory framework in African countries

The current findings highlight the growing interest among several African countries in developing and implementing regulatory frameworks to regulate the use of genome editing and gene drive technologies (Table 4). This includes the formulation of regulatory frameworks aimed at ensuring the safe and ethical use of these technologies within respective national contexts. In countries like the DRC, Eswatini, Ethiopia, Mozambique and Uganda, discussions and drafting efforts are underway to establish modern biosafety regulatory regimes. These countries recognize the need for a regulatory system tailored to address the specific challenges and opportunities presented by these technologies.

**TABLE 4:** The survey results on some African countries plan to draft or modify their existing regulations for genome editing and gene drive technologies.

Countries	Plans to modify the regulatory framework under their purview?	How modification will be made	Willingness to learn practices and experiences from others?	Readiness to share practices and experiences?
Benin	−	Through the development of specific guidelines and regulatory policies for these technologies	+	+
Ethiopia	+	Through the development of specific guidelines and regulatory policies for these technologies	+	+
Democratic Republic of Congo	+	Through the development of specific guidelines and regulatory policies for these technologies	−	−
Gambia	+	Need revision. Global Environment Facility allocated funds for an update	+	+
Mozambique	+	Through the development of specific guidelines and regulatory policies for these technologies	+	+
Tunisia	+	Through the development of specific guidelines and regulatory policies for these technologies	+	−
Uganda	+	Through the development of specific guidelines and regulatory policies for these technologies	+	+
Zambia	+	By modifying the biosafety regulations	+	+

(Mark +: denotes yes; -: denotes “No”).

### 3.5 Literature search

#### 3.5.1 Status approval of genetic engineering applications

The scientific literature provides insights into the heterogeneous landscape of approval processes for genetic engineering applications across African countries. While some nations have established regulatory frameworks for authorizing GMOs, including for research and development, CFTs, open field trials activities, and importing for the direct use of GM products, others are still in the developmental stages of such frameworks (Table 5).

**TABLE 5:** Status on authorization for genetic engineering applications in African countries according to scientific literature.

Country	Stages of development					References
	Laboratory research	CFTs	Open-field cultivations	Pre-market authorization	Importing for direct use	
Angola	+	−	−	−	−	• Muzhinji and Ntuli, (2020) • Angola succeeds in the Application of Biotechnology in Agriculture (Digital, 2022)
Benin	−	−	−	−	−	• GM crops are officially banned in Benin • There is a legal vacuum reflected by the lack of an organizational framework for regulating the production, commercialization, and importation of GM crops (Houdegbe et al., 2018)
Burkina Faso	+	+	+	+	+	• A “non-gene drive male-biased” application was authorized in July 2021 by the ANB (National Biosafety Agency) for import (Pablo, 2023) • However, no more genetically modified mosquitoes released in 2021 (Pablo, 2023) • The first phase took place in July 2019 with the release of 6,400 transgenic mosquitoes in Bana (a rural village in the center west of the country) (Vekcha, 2022) • Cowpea with GM insect resistance undergoing confined field trial (Akinbo et al., 2021) • Commercial release of Bt-cotton but put on hold in 2016 due to decision to terminate the commercial registration of GM cotton • CFTs of several GM crops — cassava, cowpea, banana, and Irish potato - are taking place (Wadvalla, 2022)
Botswana	−	−	−	−	−	Muzhinji and Ntuli (2020)
Comoros	+	−	−	−	−	Muzhinji and Ntuli (2020)
DRC	+	−	−	−	−	• Lack of adequate legislation to regulate and approve the import and to monitor the introduction of GMOs and synthetic biology products (Otono et al., 2020)
Eswatini	+	+	+	+	+	• Approved GM crop application (Wadvalla, 2022) • The commercial release of transgenic insect resistant Cotton has been approved (Akinbo et al., 2021)
Ethiopia	+	+	+	+	−	• Developing transgenic Enset resistant to bacterial wilt (Conrow, 2018) • Development of lodging resistance in tef (*Eragrostis tef*) (Beyene et al., 2022) • Insect-resistant and drought tolerant Maize under confined field trials (Akinbo et al., 2021) • TELA Maize, (drought tolerance plus insect resistance (Conrow, 2018; ABNE, 2022) • Open cultivation for Bt cotton (ABNE, 2022) • Approved commercial cultivation for insect-resistant, Bt cotton hybrids (Conrow, 2018; Gebretsadik and Kiflu, 2018; Terefe, 2018; Komen et al., 2020)
Gambia	−	−	−	−	−	−
Ghana	+	+	+	−	−	• Nitrogen and water-use-efficient (NEWEST) rice and genetically modified cowpea (Baffour-Awuah, 2022) • CFTs of several GM crops — cassava, cowpea, banana and Irish potato are taking place in Ghana (Wadvalla, 2022) • These two transgenic crops have gone through various stages of evaluation and field trials (Baffour-Awuah, 2022) • An application for commercial release has been requested and invited public comments to make a final decision on the request (Baffour-Awuah, 2022)
Kenya	+	+	+	+	+	• The National Biosafety Authority (NBA) has reviewed over 28 contained use applications (Njiraini, 2020) • NBA has approved confined field trials (Njiraini, 2020) • CFTs of several GM crops — cassava, cowpea, banana, and Irish potato - are taking place (Wadvalla, 2022) • NBA has reviewed two environmental release applications for Bt cotton and Bt maize (Njiraini, 2020) • Drought-tolerant maize approved for National performance trials (NPTs) (Komen et al., 2020) • NBA approved GM cassava resistant to brown streak virus disease (CBSD) for environmental release (Maina, 2022c) • The importation of White transgenic Maize (Maina, 2022a)
Madagascar	−	−	−	−	−	• No research and development, and no confined field trial (Muzhinji and Ntuli, 2020)
Malawi	−	+	−	+	−	• Bt cowpea and virus-resistant bananas are undergoing confined field trials (Malawi Progresses in GM Crop Trials - Chaweza (2020)) • Commercial release of transgenic insect resistance of Cotton approved
Mauritius	+	−	−	−	−	• Transgenic Sugarcane research is ongoing (Muzhinji and Ntuli, 2020)
Mozambique	+	+	−	−	−	• Conducting confined field trials for drought tolerant maize (Muzhinji and Ntuli, 2020) • CFTs of several GM crops — cassava, cowpea, banana, and Irish potato - are taking place (Wadvalla, 2022)
Namibia	−	−	−	−	−	• No transgenic crops are approved for environmental release (Muzhinji and Ntuli, 2020)
Nigeria	+	+		+	+	• Several transgenic crop research undergoing • Several transgenic crops, such as Cassava, Sorghum and Maize, are undergoing CFTs research • CFTs are completed for TELA maize (Maina, 2022) • National varieties release committee plans to evaluate TELA maize for approval before it is made commercially available for the 2023 season (Maina, 2022) • General release and submission for variety registration for insect-resistant cowpeas (Komen et al., 2020; ABNE, 2022) • TELA is first transgenic maize variety to move toward adoption (Maina, 2022)
South Africa	−	−	−	+	+	• Farmers are commercially growing TELA maize (drought- and insect-tolerant) (Maina, 2022) • Herbicide-tolerant soybean commercially released (Muzhinji and Ntuli, 2020) • Importation of transgenic Bt white maize and TELA maize authorized (Maina, 2022)
Tanzania	+	+	−	−	−	• Transgenic virus-resistant cassava under development • Researchers conducting CFTs for drought-resistant maize and virus-resistant cassava (Muzhinji and Ntuli, 2020)
Tunisia	+	−	−	−	−	• Does not import any GM food or feed (banned) (FAO, 2019)
Uganda	+	+	+	+	−	• Weevil-resistance sweet potato and herbicide-tolerant soybean are under lab research (Ghislain et al., 2018; ABNE, 2022) • Has approved field experiments involving transgenic crops (Zawedde et al., 2018) • RNAi-mediated resistance to Cassava brown streak disease (CBSD) (Odipio et al., 2013; ABNE, 2022) • CFTs of several GM crops — cassava, cowpea, banana, and Irish potato - are taking place (Wadvalla, 2022) • Several transgenic crops are undergoing multiplication trials (Zawedde et al., 2018) • For some transgenic crops, multi-location trials are completed and awaiting biosafety decision regarding environmental release (Zawedde et al., 2018)
Zambia	+	−	−	−	−	• Lab research is authorized but no CFTs, open field trials, and environmental release authorization for transgenic crops (Muzhinji and Ntuli, 2020)

(Mark “+”: denotes the availability of scientific evidence for the approval of genetic engineering application at various stages; “-”: denotes “no scientific evidence available for approval of genetic engineering application at various stages).

#### 3.5.2 The literature finding on the regulatory landscape of genome editing and gene drive in Africa

Literature findings concerning the regulatory frameworks surrounding genome editing and gene drives in African countries are critical for understanding the various laws, policies and guidelines governing these technologies on the continent. Recent studies have shed light on how different African countries approach genome editing and gene drive regulation. African countries have developed various types of regulatory approvals for genome-edited product usage, i.e., laboratory research and development, confined/small scale field trials, open scale field releases, and multiplication field trials for agronomic performance evaluation and commercialization in African countries (Table 6).

**TABLE 6:** The literature findings analyzed from academic journals on the status of genome editing and gene drive technologies in African countries.

Country	Gene Editing				
	Laboratory Development and Biosafety regulatory framework development status^a^	Contained trial^b^	Confined trials/Small-scale field release^c^	Open-scale field release^d^	Multiplication field trials/Large-scale controlled field releases^e^
Burkina Faso	• Started considering developing genome-editing policies (Tripathi et al., 2022) • Community and stakeholder engagement work done (Toe et al., 2022)	−	−	−	−
Eswatini	• Lab facilities are organized • Started considering developing genome-editing policies (Tripathi et al., 2022)	−	−	−	−
Ethiopia	• Started considering developing a genome-editing regulatory framework (Tripathi et al., 2022)	• Lodging resistance in Teff successfully developed using CRISPR/Cas9-based genome editing (Beyene et al., 2022) • CRISPR-Cas9 has potential to be used for Enset to combat wilting (Merga et al., 2019)	−	−	-−
Ghana	• Lab facilities are organized and started considering developing genome-editing policies (Tripathi et al., 2022)	−	−	−	−
Kenya	• NBA published guidelines suitable for regulating genome-edited organisms and products (ISAAA, 2022) • CRISPR/Cas9-based research in yam (Dioscorea spp.) to induce site-specific disruption of PDS gene developed (Syombua et al., 2021)	+	+	+	• Researchers applied for approval of multiplication trials • Six applications approved for contained use
Malawi	• Genome Editing Guidelines approved, joining Nigeria and Kenya. (ISAAA, 2022)	−	−	−	−
Nigeria	• Nigeria has become first African country to publish national biosafety guidelines for genome editing regulation (USDA, 2021; Tripathi et al., 2022)	• Guidelines are issued for gene editing of plants, animals, and microorganisms, covering containment, field trials, commercial/general release and imports for food or feed (Tripathi et al., 2022)	+	+	• Approval for multiplication trials for genome editing products on a case-by-case basis (Tripathi et al., 2022)
South African	• Currently developing regulatory policies for genome editing (Tripathi et al., 2022)	−	−	−	−
Sudan	• Lab facilities are developed • Started considering developing genome-editing policies (Tripathi et al., 2022)	−	−	−	−
Uganda	• Drafting guidelines for gene-edited crops through NBC support • Contained facility laboratory to be established for contained trials of male sterile mosquitoes • No field trials with gene-edited crops yet (Ongu et al., 2023)	−	−	−	−
Zimbabwe	• Lab facilities are organized and started developing genome-editing policies (Tripathi et al., 2022)	−	−	−	−

Country	Gene Drive				
	Laboratory research	Contained trial	Confined trials/Small-scale studies	Open-scale field release	
Burkina Faso	• Researchers anticipate the release of genetically engineered mosquitoes to initiate gene drives aimed at reducing insect populations (Swetlitz and Stat, 2024) • Existing guidelines for non-gene drive mosquitoes can provide an overall framework for gene drive technology on case-by-case basis (Toe et al., 2022)	−	−	• Target Malaria plans to conduct the first field trials of gene drive mosquitoes in partner countries like Uganda, Burkina Faso or Ghana within 5–10 years (Scudellari, 2019)	
Ethiopia	• No research at the laboratory level • African Union declared commitment to invest in the development and regulation of gene-drive technology (AUDA-NEPAD, 2023)	−	−	−	
Kenya	• No gene drive research at the laboratory level yet	−	−	−	
Nigeria	• In August 2019, the “National Biosafety Management Agency (NBMA) Act 2015” accepted emerging agricultural biotechnologies - gene drive, gene editing, and synthetic biology (NBMA, 2015) • A journalist was trained on the controversies surrounding “gene drives” research, aiming to equip journalists with the necessary understanding of the issues surrounding gene drive (Omoniyi, 2024)	• A person or institution shall not carry out gene drive, gene editing, and synthetic biology except with the approval of the Agency (NBMA, 2015)	−	−	
Uganda	• The GMO Bill, with little mention of gene drive, has been one of the most debated bills in Uganda’s history (Bendana, 2020) • The President refused to approve the 2018 GMO Bill due to ecological and health concerns (Duba, 2021)	−	−	• No gene drive organism has yet been tested in the field (Yao et al., 2022) • Uganda may host the first field trials of gene drive mosquitoes for malaria control (Scudellari, 2019; Hartley et al., 2024) • Decision on whether to release gene drive mosquitoes is yet to be made (De Graeff et al., 2021)	

(Mark “+”: denotes availability of scientific evidence indicating approval for genome editing and gene drive application at various stages; “-”: denotes no scientific evidence available for approval for genome editing and gene drive application at various stages.

^a^ Indicates the progress in laboratory research and the status of biosafety regulatory frameworks that guide the development of genome editing and gene drive technologies.

^b^ Refers to trials conducted in highly controlled environments, such as greenhouses or laboratories, where genome-edited organisms are kept isolated to prevent unintended environmental exposure or risks.

^c^ Highlights small-scale field trials conducted under regulated conditions, where genome-edited organisms are tested outdoors in confined areas.

^d^ Represents large-scale, less confined field trials where genome-edited organisms are released into the environment under supervision.

^e^ Shows the status of large-scale controlled field releases, where genome-edited organisms are grown in significant quantities.

## 4 Discussion

In light of the diverse regulatory landscapes, approval processes and approaches toward genome editing and gene drive, the discussion section explores the implications and broader significance of these findings. By synthesizing the results of the literature search and survey analyses, this section aims to elucidate key insights, identify challenges, and propose avenues for future research and policy development concerning genetic engineering applications and biotechnology regulation across African countries.

### 4.1 Authorization of genetic engineering applications in Africa

The regulatory framework landscape for genetic engineering applications in Africa varies significantly between countries. While some countries have enacted comprehensive regulations for the use of GMOs, others have more outdated regulatory frameworks. Several African countries have biosafety regulatory frameworks that can approve genetic engineering applications in CFTs, open field trials, commercialization and importation for use as food or feed, while many African countries have no experience in authorizing CFTs or even have a biosafety regulatory framework at all (ABNE, 2023).

The survey findings (Table 1) and literature review (Table 5) reveal both consistent patterns and new insights. In several countries, such as Eswatini, the Democratic Republic of Congo (DRC), Kenya, Mozambique, and Nigeria, the approval processes for genetic engineering applications appear to be interrelated and mutually supportive. However, the survey also introduces new information not previously documented in the literature. For instance, Ethiopia and Ghana have authorized the full spectrum of genetic engineering applications, including the importation of GM plants for direct use. Additionally, Zambia has approved genetic engineering for R&D, CFTs, and the importation of GM crops for food and feed—details that have not been reported in earlier studies. In cases where survey data were unavailable, such as Burkina Faso, Malawi, and Mauritius, the literature review helped fill gaps and validate the survey results.

Eleven countries (Burkina Faso, Egypt, Eswatini, Ethiopia, Ghana, Kenya, Malawi, Nigeria, South Africa, Sudan, and Zambia) have approved GM crop applications, although Zambia has yet to establish biosafety laws regulating GMOs (ABNE, 2022; ISAAA, 2024 as reviewed in Mmbando, 2024). The other ten countries have conducted field trials and approved the commercial cultivation of GM crops (Mmbando, 2024). Despite several field trials and commercial release authorizations, African countries continue to bear the reputation of being slow adaptors of GM technologies, mainly attributed to overly restrictive biosafety regulatory frameworks (Ongu et al., 2023) and political challenges (Komen et al., 2020) often exacerbated by anti-GMO activism which slowed or halted the adoption of biosafety legislation (Entine, 2022). These challenges hinder the testing and adoption of new crop varieties, including those developed by genome editing or other advanced technologies, which aim to enhance income and reduce the environmental impact of agriculture (Komen et al., 2020). Notably, African researchers are working tirelessly to develop biotechnology products so that Africa is no longer a battleground for adopting GMOs (Muthie, 2022).

According to data retrieved from ABNE in August 2023, countries such as Ghana, Tanzania, Mozambique, and Uganda have experience approving CFTs. Additionally, Burkina Faso, Nigeria, Sudan, Ethiopia, Kenya, Malawi, and Eswatini have approved commercial cultivation of GM crops. In particular, Eswatini, Ghana, Kenya and Nigeria are the front liners in the development and maturation of national biosafety laws and, hence, they have authorized the genetic engineering applications for several activities (Table 1). In contrast, Benin has not approved any commercial release or importation of GMOs. Despite the existence of relevant institutions in Benin, a legal vacuum appears to persist, reflected in the absence of an organizational framework for regulating the production, commercialization and importation of GMOs (IOBC-WPRS, 2023).

CTFs have been approved for various crops in several African countries: maize, cotton, sorghum, cassava and sweet potato in Kenya; cassava, cotton, cowpea, rice and soybean in Nigeria; banana, cassava, maize, potato and rice in Uganda, and; cotton, cowpea, banana and plantains in Malawi (Komen et al., 2020) (Table 5). Additionally, CFTs for GM crops — such as cassava, cowpea, banana and Irish potato are taking place in Mozambique, Kenya, Uganda, Ghana, Burkina Faso and Rwanda (Wadvalla, 2022). In contrast, Zambia, Tunisia and the DRC have not yet authorized CFTs, according to ABNE. This lack of authorization could be due to the lack of appropriate legislation to regulate the importation of GMOs and to monitor their introduction (Otono et al., 2020) (Table 5). The DRC is currently considering revising or strengthening its biosafety framework (Otono et al., 2020).

Ghana stands out as a West African country that have approved genetic engineering applications for laboratory research, CFTs, open-field trials and commercial cultivation. In 2012, the National Biosafety Committee in Ghana approved multi-locational trials for insect-resistant GM cotton after accepting data from CFTs conducted previously in Burkina Faso (Komen et al., 2020). Genetically modified nitrogen- and water-efficient rice and pod borer-resistant cowpeas have not been commercialized yet, although they have undergone various stages of evaluation and field trials (Baffour-Awuah, 2022).

In general, most African countries have not yet fully overcome the challenges associated with GMOs. These hurdles may pose difficulties in adapting to new regulatory frameworks and governance of emerging technologies such as genome editing and gene drive (Masehela and Barros, 2023).

### 4.2 Status of genome editing and gene drive regulatory frameworks in Africa

Genome editing technology is increasingly attracting the interest of African researchers due to its potential to address agricultural, health and environmental issues. To fully benefit from genome editing, there are calls that products developed through this technology must be subjected to reasonable, science-based regulation (Entine et al., 2021). Globally, while some countries have quickly adapted or amended their legislation to support the use of genome editing, others are in the initial stages of policy development (Jenkins et al., 2021). However, some countries still categorize all organisms derived through genome editing as GMOs (Hundleby and Harwood, 2022).

In Africa, the regulatory landscape is varied. Some countries have enacted regulatory frameworks despite not yet utilizing crop genetic engineering technology, while others have already conducted field trials and are beginning to authorize GM crops for cultivation (Komen et al., 2020; ABNE, 2022). This study found that few African countries have developed specific regulatory approaches tailored to genome-editing. For instance, the survey analysis (Table 2) highlights that countries like Benin, Eswatini, Kenya, Nigeria, and Uganda have established governance frameworks for genome editing, an observation also supported in the literature (Meeme, 2021; Genetic Literacy Project, 2021; Wadvalla, 2022; Buchholzer and Frommer, 2023). Additionally, Burkina Faso and Ghana have begun considering developing policies for genome editing (Tripathi et al., 2022) (Table 6).

Nigeria was the first African country to publish its regulatory frameworks on genome editing in December 2020, followed by Kenya, which published its frameworks in February 2022, an important milestone regulatory development for genome editing in Africa (ISAAA, 2022). Rather than amending its biosafety act, Kenya’s National Biosafety Authority (NBA) opted to develop a guideline on genome editing (Komen et al., 2020).

Malawi also approved its regulatory frameworks for genome editing in August 2022 (ISAAA, 2022), and Burkina Faso has validated the national regulatory frameworks, which are awaiting final approval and publication (AUDA-NEPAD, 2023). Burkina Faso is, therefore, expected to become the fourth African country to approve and publish national biosafety regulatory frameworks for genome editing. Eswatini, too, has made significant progress in establishing regulations for genome editing (Meeme, 2021).

Although Benin, Eswatini and Uganda have not yet formally published their regulatory frameworks for genome editing and gene drive, their existing biosafety regulations could be applied to oversee these technologies. This approach is similar to the EU regulatory framework, where existing biosafety regulations are applied without amendments, requiring prior government safety approval, which is the most stringent regulation for genome editing products (Tachikawa and Matsuo, 2023). Furthermore, the survey findings show that the regulatory frameworks in Benin, Eswatini, and Uganda include provisions for genome editing and gene drive technologies, deregulating SDN-1 and SDN-2 techniques while regulating SDN-3 on a case-by-case basis (Table 2). However, a detailed investigation into the regulatory status of genome editing and gene drive technologies in these African countries remains largely unexplored in the scientific literature. On the other hand, in Burkina Faso, Ethiopia, Ghana, South Africa, Sudan, and Zimbabwe, lab facilities have been developed and initiated developing genome editing policies (Tripathi et al., 2022) (Table 6). Moreover, survey results from Burkina Faso, Cameroon, Kenya, Liberia, Mali, Nigeria, South Africa, and Uganda claimed that there are ongoing research activities on Gene Drive Modified Mosquito (GDMM) in the laboratory phase and emphasized the importance of a regulatory framework (Finda et al., 2023). Several African countries, including the DRC, Ethiopia, Gambia, Ghana, Mozambique, Tanzania, Tunisia, and Zambia, lack biosafety regulatory frameworks governing genome editing and gene drive technologies. However, Ethiopia, Ghana, Sudan, South Africa and Zimbabwe have begun considering the development of such frameworks (Jenkins et al., 2021; Tripathi et al., 2022; Africa, 2022). The absence of clear regulatory frameworks in these countries poses a challenge to the development, adoption and implementation of genome editing and gene drive technologies. In response, several African countries have shown interest in revising or adapting their existing regulatory frameworks to accommodate these emerging biotechnologies (Meeme, 2021).

The survey found that biosafety experts and regulators in the DRC, Gambia, Ghana, Mozambique, Zambia and Tanzania are interested in amending their biosafety regulatory frameworks to regulate genome editing and gene drive products effectively. By doing so, these countries aim to fully leverage the potential of genome editing and gene drive technologies.

### 4.3 Challenges in implementing and harmonizing biosafety regulatory frameworks in Africa

African countries face significant challenges in developing and implementing regulations for genome editing and gene drive technologies. Addressing these gaps is crucial for ensuring the responsible and ethical use of these technologies. Our survey analysis identified several key challenges, including a lack of public trust, opposition, and moral concerns regarding genome editing and gene drive technologies. Public trust is critical to the successful adoption and realization of the benefits of these technologies on the African continent (Masehela and Barros, 2023).

The survey revealed that limited resources, a lack of trained and skilled expertise, insufficient public awareness, and public resistance or scepticism had hindered the implementation of national biosafety regulations for genome editing and gene drive technologies. Masehela and Barros (2023) also noted that the lack of trained technical expertise, inadequate intellectual property rights infrastructure and various concerns on genome editing products and unsupportive government leadership remain prevalent on the African continent. Similarly, Maruta et al. (2023) highlighted that insufficient infrastructure, training, capacity building and financing affect efforts to strengthen biosafety in African nations. Enhancing expertise for the development of regulatory frameworks for genome editing or gene drive technology could increase the legitimacy of the overall assessment process (Adenle et al., 2013).

Our survey analysis further indicates that negative public perception is a significant barrier, primarily due to the perceived lack of relevant data about the technologies as they are still in their early stages of development, together with fears about their potential unintended consequences or misuse. Abkallo et al. (2024) reported that mistrust surrounding genome editing technology arises from ethical concerns, fears of unintended consequences and a lack of transparency in communication, which hinder its widespread acceptance and implementation in African nations. Public perception can significantly impact gene editing technology implementation; thus, both public and stakeholder acceptance is crucial for favorable regulations and deployment (Strobbe et al., 2023).

Unharmonized regulations, especially with respect to definitions and risk assessment approaches, can hinder the applications of these technologies and future trade between countries. Therefore, harmonizing the regulatory climate is essential for equitable access and international trade in technology products (Hundleby and Harwood, 2022). However, several challenges impede the harmonization of the biosafety regulatory framework in African countries. Our survey found that the lack of coordination and information-sharing among regulatory agencies, issues related to technology transfer, concerns about intellectual property rights, the absence of region-specific regulatory frameworks for genome editing, the existence of different regulatory frameworks and definitions, and difficulties in aligning different cultural, social, and ethical perspectives were cited as major obstacles to harmonizing biosafety regulatory frameworks at the regional level. This finding is supported by a report (Africa, 2016) highlighting that significantly different legislation, definitions, and regulatory approaches among countries hinder regional or international harmonization. The report emphasized the importance of cooperation and harmonization among regulators to prevent arbitrary decisions, regional and international asymmetries in scientific and technical development, and trade barriers.

To address these challenges, it is crucial for African countries to collaborate, discuss, debate and attempt to harmonize their science-based regulatory frameworks. According to the chief executive of Kenya’s National Biosafety Authority (NBA), many countries in the Global North have adopted a common approach to regulating these new technologies, so there is an opportunity for the Global South to learn from them in developing approaches for these new technologies, by promoting greater international collaboration in knowledge sharing and figuring out how to best utilize new technologies that are being developed or used in their own countries (Ladenheim, 2023). This will prepare them for the eventual approval and use of products developed using genome editing approaches (Masehela and Barros, 2023). Regulatory diplomacy can play a critical role by facilitating dialogue and collaboration, ensuring that diverse perspectives are captured in cohesive regulatory approaches (Warner and Pink, 2024). Such collaborative efforts can foster a more cohesive regulatory environment, facilitating the responsible development and deployment of genome editing technologies across the continent.

### 4.4 Challenge-driven opportunities for developing biosafety regulatory framework in Africa

Africa faces an urgent need to close the yield gap in staple crops and increase food production to feed the growing population (Tripathi et al., 2022). The challenge of producing more food with the same or less land and water, improving nutrition and helping farmers adapt to climate change is particularly acute (Searchinger et al., 2024). Climate change is predicted to negatively impact the African food system, as agriculture in the region is largely dependent on rain-fed farming and subsistence agriculture (Manners et al., 2021). Additionally, climate change may drive the expansion of disease vectors and vector-borne pathogens like dengue, Zika, and Chikungunya viruses (De Souza and Weaver, 2024), creating suitable temperature conditions for transmission beyond current limits (Samy et al., 2016; Minigan et al., 2018; Kraemer et al., 2019; Ryan et al., 2019). These challenges underscore the need to scale up research, build capacity, and develop genome editing and gene drive technologies within appropriate regulatory frameworks. Realizing the full potential of new breeding tools, such as genome editing, alongside conventional technologies is essential (Pixley et al., 2019).

To leverage these advancements, African countries must develop new regulatory frameworks or adjust existing biosafety regulatory frameworks to accommodate genome editing and gene drive technologies, perhaps drawing on models of Nigeria (USDA, 2021) and Kenya (ISAAA, 2022). In this context, adopting adaptive regulatory frameworks will enable African countries to respond more effectively to the rapid developments in genome editing and gene drive technologies while addressing public concerns and ethical considerations (Greer and Trump, 2019).

In regions with low awareness and high public resistance to genome editing and gene drive technologies, effective regulation can assure society that these technologies will not be misused. Building public trust is critical to the success and acceptance of these technologies (Masehela and Barros, 2023) while addressing concerns related to safety (Trump et al., 2023).

The regulatory challenges encountered with GM crops provide valuable lessons that can inform the development of regulatory frameworks for genome editing and gene drive technologies (Rock et al., 2023). These experiences offer an opportunity to create more robust and informed regulatory frameworks.

### 4.5 Strengthening the regulatory landscape in Africa

Our survey analysis highlights the importance of investing in training programmes and workshops for regulators and stakeholders to strengthen the regulatory framework for genome editing and gene drive technologies. Establishing partnerships with academic institutions for research and capacity building, along with increasing knowledge-sharing with international organizations and experts, emerged as key strategies to enhance the regulatory framework in African nations.

Enhancing research and development capacities through international collaboration has already shown promising results. For instance, Nigeria, Kenya and Uganda have significantly advanced their plant genome editing technology capacities through partnerships with the International Institute of Tropical Agriculture (IITA) and the Swedish University of Agricultural Sciences (SLU) (Ongu et al., 2023). Such collaborations provide a blueprint for other African countries to strengthen their regulatory landscape.

A critical element in this process is the proactive engagement of diverse stakeholders, including policymakers, scientists, the public and educators, in open and accessible dialogues concerning the scientific principles, potential benefits and risks associated with genome editing (Abkallo et al., 2024) may ensure a more inclusive and transparent regulatory process. Public participation, transparency and accountability are crucial elements in policy development and agenda-setting, playing a significant role throughout the regulatory pathway (Nielsen et al., 2021). Without the bottom-up regulatory structures involving public engagement and the pursuit of people-centered, measures to hold researchers accountable for unethical research practices could be weak (Barbosa et al., 2021). Incorporating this approach can help address public concerns, build trust and ensure that the regulatory frameworks are comprehensive and well-informed by a wide range of perspectives and expertise.

## 5 Conclusion

The regulatory landscape for genome editing and gene drive technologies in Africa is diverse and evolving. Our study has highlighted significant disparities in the development and implementation of biosafety frameworks across different countries. While nations such as Nigeria and Kenya have made notable strides in establishing regulatory frameworks specific to genome editing, others are still in the preliminary stages of policy formulation. The findings indicate that the lack of clear, harmonized regulations poses challenges to the adoption and application of these technologies. Additionally, issues such as limited resources, inadequate technical expertise and public scepticism further complicate the regulatory environment.

Despite these challenges, there is a strong interest among African countries to harness the potential of genome editing and gene drive technologies to address pressing agricultural, health and environmental issues. The successful implementation of these technologies will require comprehensive regulatory frameworks that are science-based, context-specific and adaptable to emerging innovations. It is also evident that building public trust through transparent communication and stakeholder engagement is crucial for the acceptance and success of these technologies.

## 6 Actionable recommendations

Based on the findings of this study, the following recommendations are proposed to strengthen the regulatory frameworks for genome editing and gene drive technologies in Africa:

1. Build Capacity and Provide Training: Continue offering comprehensive training programmes and workshops for regulators and stakeholders. While traditional programmes remain important, also explore innovative approaches such as inter-agency consultations and internships in countries with established practices. These initiatives will equip local regulators and technical advisors with the practical experience and knowledge needed to contribute to informed decision-making and effective regulatory oversight.
2. Foster International Collaboration: Promote partnerships with international organizations, academic institutions and experts to facilitate knowledge transfer and technical assistance. By fostering international cooperation, countries can benefit from shared expertise in developing robust regulatory frameworks. Emphasize regulatory diplomacy to navigate global advancements, ensuring Africa’s regulatory systems remain aligned with global developments.
3. Adopt Best Practices: Incorporate best practices from countries with established regulatory frameworks, such as Nigeria and Kenya. While leveraging these successful models, tailoring them to the unique contexts and needs of other African nations is crucial. This ensures that the regulatory process becomes more relevant and adaptable to each country’s specific challenges and opportunities.
4. Develop Context-Specific Regulatory Frameworks: Formulate flexible regulatory frameworks that can accommodate ongoing technological advancements. These frameworks should address local ethical, social, and environmental considerations. This approach can help empower African countries to develop regulations that reflect their national aspirations together with their specific context and challenges.
5. Harmonize Approaches that Underpin Regulatory Decision-Making: Align key regulatory decision-making components, such as definitions and risk assessment methodologies, across African countries. This alignment can reduce inconsistencies and asymmetries between national regulatory systems while preserving flexibility for local adaptations. Additionally, recognize technical data, analyses, and assessments from foreign or neighboring regulatory authorities, reducing duplicative work and increasing the efficiency of regulatory authorizations. This will help lower costs and create smoother, faster approval processes.
6. Encourage Stakeholder Involvement: Engage a broad range of stakeholders, including scientists, policymakers, industry representatives and the public (including indigenous people and local communities). Ensuring their active participation in the regulatory process can lead to more comprehensive, widely accepted regulatory frameworks. Inclusive consultations help bring diverse perspectives to the table, improving the quality and transparency of regulations.
7. Engage the Public and Educate: Implement innovative education campaigns to raise public awareness about the potential benefits and risks of genome editing and gene drive technologies. Transparent and accessible communication can mitigate public skepticism, foster trust in the regulatory process, and support informed decision-making. Ensuring that the public understands the science and the risk management in place would lead to greater acceptance of these technologies.

By implementing these recommendations, African countries can develop robust and adaptive regulatory frameworks that support the safe, ethical and beneficial use of genome editing and gene drive technologies. This proactive approach will help enable the continent to address critical challenges in agriculture, health and environmental sustainability, ultimately contributing to improved livelihoods and economic development.

## Data Availability

The original contributions presented in the study are included in the article/supplementary material, further inquiries can be directed to the corresponding authors.
